# Perspectives on Eco-Friendly Food Packaging: Challenges, Solutions, and Trends

**DOI:** 10.3390/foods14173062

**Published:** 2025-08-30

**Authors:** Paula Fernanda Janetti Bócoli, Vitor Emanuel de Souza Gomes, Amanda Alves Domingos Maia, Luís Marangoni Júnior

**Affiliations:** 1 Department of Food Engineering and Technology, School of Food Engineering, Universidade Estadual de Campinas (UNICAMP), Campinas 13083-862, Brazil; p035156@dac.unicamp.br (P.F.J.B.); v168392@dac.unicamp.br (V.E.d.S.G.); 2 Packaging Technology Center, Institute of Food Technology, Campinas 13070-178, Brazil

**Keywords:** food packaging, biomaterials, circular economy, material recyclability, innovative design

## Abstract

The development of sustainable packaging in the food industry is essential to meet the growing demand for more environmentally friendly practices and to contribute to material circularity and solid waste reduction. In this context, this review explores the main categories of sustainable packaging in the food industry, including recyclable, reusable, biodegradable, and compostable packages, highlighting the materials used, their characteristics, advantages, and limitations. Furthermore, it discusses innovations that combine convenience and safety with lower environmental impact, such as the use of biopolymers, and nanomaterials that extend food preservation, enhance properties, and enable broader application. The adoption of these technologies can reduce dependence on fossil-based plastics and minimize environmental impacts, although challenges remain, such as economic viability, regulatory standardization, and consumer acceptance. Additionally, the review addresses difficulties related to recycling and reverse logistics, emphasizing the need for a joint effort among companies, governments, and consumers to promote a more sustainable food system. Thus, the research highlights the importance of innovation and collaboration in developing viable solutions that reconcile sustainability, food safety, and efficiency in the packaging industry.

## 1. Introduction

Food packaging plays an essential role in the dynamics of the global economy, going far beyond providing mere containers for food. It serves to protect, preserve, and ensure the integrity of products from production to final consumption [1]. In this process, packaging guarantees the quality and safety of foods, acting also as the first line of defense against chemical, physical, and microbiological contamination, directly safeguarding consumers’ health [2]. The materials traditionally used for food packaging include petroleum-based plastic polymers, glass, metals, and paper, each offering specific advantages such as flexibility, strength, ease of production, efficient storage, and versatile transportation [3]. These options, although effective, also have their limitations, especially in the context of growing environmental concerns.

In response to the environmental issues caused by the massive use of conventional packaging and the depletion of natural resources, the industry has sought to innovate and develop more sustainable alternatives. Research and projects focused on creating packaging that promotes safety, traceability, and sustainability are gaining prominence, aiming to establish more responsible and less environmentally impactful food supply chains [4,5]. The priority is to reduce losses and waste throughout the entire supply chain while minimizing the harmful effects on the planet, a trend that is gaining momentum among manufacturers, consumers, and regulators [6,7,8,9].

Assessing the environmental and economic impact of packaging throughout its entire life cycle has become a key requirement for companies seeking to strengthen their commitment to sustainability. In this context, interest in recyclable materials and eco-friendly solutions is intensifying, also driven by the desire to better understand consumer perceptions regarding the environmental impacts caused by packaging [10]. For these innovations to succeed, it is crucial to understand the benefits, challenges, and impacts of sustainable systems throughout the entire life cycle of packaging, considering attributes such as biological origin, recyclability, and biodegradability, which are recognized as essential for reducing environmental footprint [11,12,13,14].

Among the most promising sustainability strategies, the practice of reusing packaging stands out. This approach can come from consumers who wash and reuse containers for other purposes, as well as from manufacturers and retailers who collect, clean, and reuse their own containers, promoting resource savings and waste reduction. Recycling, in turn, plays a vital role by enabling materials to be reintegrated into the production chain, fostering an efficient circular economy. Additionally, strategies such as energy recovery or reclaiming components from discarded materials are gaining traction, contributing to the reduction of environmental impact [15].

Despite advances, investment in sustainable solutions still faces significant obstacles. Many companies use recycled materials as a marketing strategy or for technological and design reasons, without a genuine commitment to sustainability [16,17,18]. Although this practice can enhance corporate image and demonstrate environmental awareness, it ultimately does little to address the core issue of increasing solid waste volumes. This underscores the urgent need for more committed and responsible approaches to natural resource management. Based on this overview, the present review aims not only to synthesize the main sustainability strategies applied to the food packaging sector but also to highlight research gaps and opportunities for innovation that still remain open. In this regard, the work proposes to critically analyze recent advances and existing limitations regarding recyclable and reusable packaging, as well as to explore the emerging role of innovative materials, such as biopolymers and nanomaterials, in the development of more efficient, safe, and sustainable packaging systems. Thus, the goal is to provide a clear view of current contributions, the barriers that need to be overcome, and future directions for the development of sustainable food packaging.

## 2. Categories of Sustainable Packaging: Reusable and Recyclable

Packaging plays a crucial role in daily life by ensuring the preservation of products during handling, transportation, and storage, as well as extending their shelf life, aiding communication, and boosting sales [4,19,20]. The choice of packaging material involves considering its properties and the product’s protection needs, but environmental concerns are also an essential factor. Among the available materials, plastics are the most commonly used due to their lightweight nature, low cost, versatility, and aesthetic variety, allowing for wide applicability [21,22].

**Figure 1:**
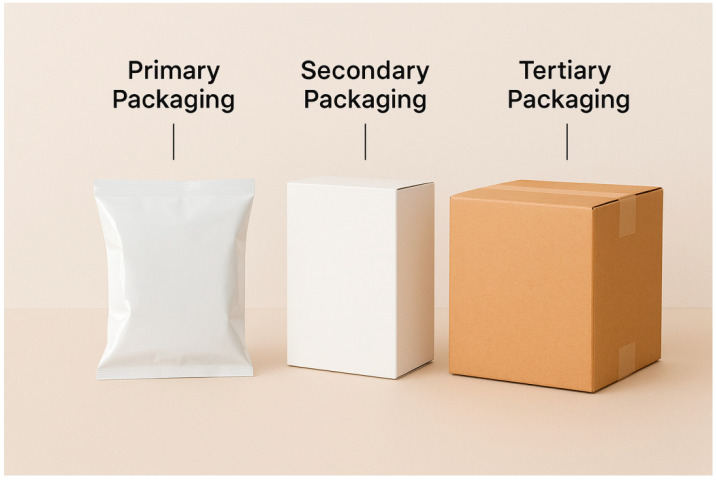
Types of packaging: primary, secondary, and tertiary.

Packaging is classified into primary, secondary, and tertiary categories. The primary packaging comes into direct contact with the product and is handled by the consumer. The secondary packaging groups primary units, facilitating transportation and organization at the point of sale. The tertiary packaging is intended for the storage and handling of products that are already contained in secondary packaging [23,24]. Figure 1 illustrates the three categories according to their classification.

Based on its destination after use, packaging can be disposable, sent directly for recycling or landfills after a single use, which represents a significant environmental impact due to the volume discarded. Alternatively, returnable packaging, such as beverage bottles, re-enters the packaging chain or is reused by the consumer, thus classified as reusable. Recyclable packaging is equally important, as it allows for the reuse of material in the production of new packaging, reducing the consumption of natural resources [22,25,26].

In this regard, packaging materials can be considered sustainable if they contribute to the reduction of virgin resource usage and if post-consumer materials are recyclable or reusable, thereby promoting a circular economy [21]. Sustainable packaging fosters a system where materials are continuously repurposed, minimizing waste and the demand for new resources, which benefits both the environment and the economy.

### 2.1. Reusable Packaging

The concept of reuse is detailed in Article 3 of the European Waste Directive of 2008 (2008/98/EC), which defines it as “any operation in which products or components that are not considered waste are used again for the same purpose for which they were originally designed” [27]. This concept is essential for the circular economy, which emphasizes prolonging the lifespan of products and minimizing waste. Reuse encompasses a wide range of products, from small items like bottles and bags to large equipment, and it is crucial to understand the different categories of reuse, as each has distinct levels of environmental impact [28].

Reusable packaging has a long history of application across various sectors and represents a significant opportunity to maximize the functionality of materials while simultaneously reducing the demand for new resources. This practice not only decreases the consumption of raw materials but also lowers the carbon emissions associated with the production process, as it requires less energy and resources than the manufacturing of new packaging [22,29]. Reuse allows materials, especially plastics, to find new pathways to add value instead of being improperly discarded. However, the implementation of reusable systems faces barriers, such as organizational and economic issues, that hinder large-scale adoption, especially when considering the reduction of virgin polymer use in food packaging [30].

Reusable packaging can be organized into distinct categories based on the characteristics of the refilling systems they use [29,30]. For example, many consumers use packaging they already have when purchasing bulk products at establishments such as supermarkets and local markets, where they can acquire items like cereals, oils, and personal hygiene products. In other cases, the original packaging is refilled with products that are typically packaged in a way that generates less waste. Refilling can occur in different ways: for instance, the product may be transferred directly into the original packaging for cosmetic or hygiene items; in other cases, the product is fitted into the original packaging, as happens with makeup refills; or there are systems where products are diluted or concentrated within the original packaging, as seen with flavored waters.

**Figure 2:**
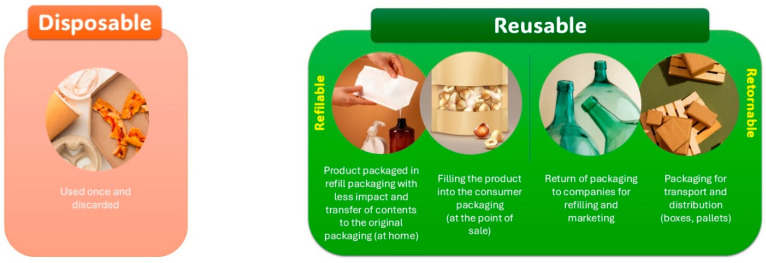
Categorization of disposable and reusable packaging.

Additionally, returnable packaging, especially in the beverage sector, is a widely recognized example of reuse. In this context, empty bottles are returned to retail points, where beverage companies collect and clean them, preparing these containers for the reuse cycle. Another type of reusable packaging involves solutions used for the transportation and distribution of products. Boxes, pallets, and other packaging systems can be used multiple times during transport, both in the B2C (business-to-consumer) and B2B (business-to-business) models, which reduces the need for new packaging production and lessens the environmental impact associated with transportation [26,31]. Figure 2 illustrates the categorization of disposable and reusable packaging.

The transition to a reusable packaging system represents a substantial challenge that must be jointly addressed by producers, retailers, and consumers. This is because not all supply chains and distribution systems are suitable for the implementation of reusable packaging. Furthermore, the organization of such a system may not yield significant environmental benefits if it is not effectively planned. To ensure a successful transition, comprehensive development is needed, which includes defining specifications for primary and secondary packaging, creating efficient monitoring systems, implementing effective reverse logistics, and establishing a robust organization of services [29].

### 2.2. Recyclable Packaging

Recycling is defined as the process of recovering materials from a product at the end of its useful life to be reused in new applications, supporting a circular economy and minimizing waste disposal [19,28]. This practice opposes traditional disposal methods, offering environmental, economic, and social advantages such as job creation, energy savings, reduction of environmental impacts, including decreased greenhouse gas emissions, and the production of new products with quality similar to that of virgin raw materials [19].

**Figure 3:**
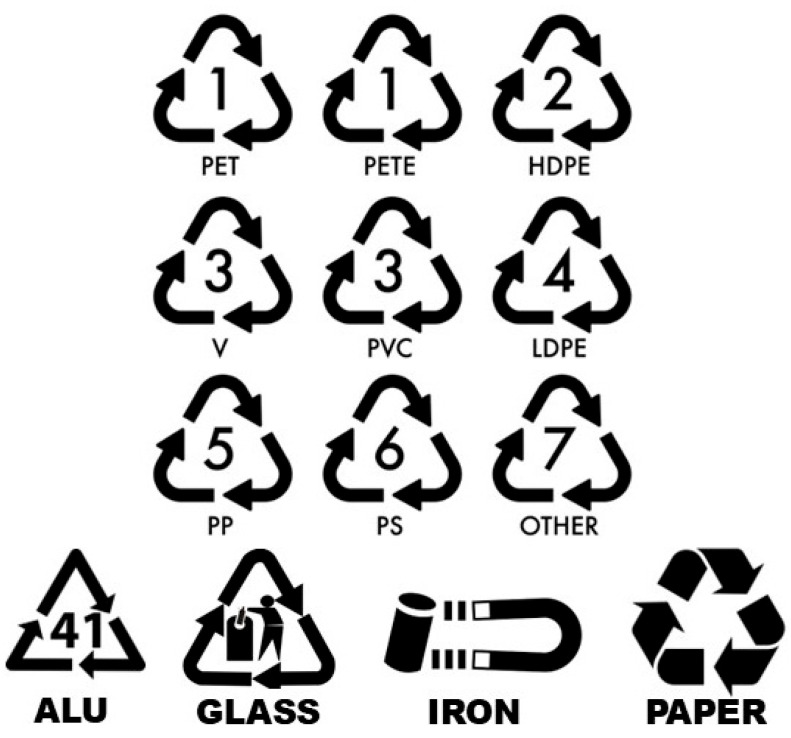
Recycling symbols of materials. Source: Author (Freepik).

The recycling of packaging involves four main steps: collection, separation, reprocessing, and marketing [19]. The quality of the recycled material directly impacts the efficiency of the process, the integrity of the final product, and its appropriate application [22]. However, one of the most common challenges is consumer behavior, particularly improper waste disposal, which hampers the success of recycling efforts and harms the environment [19]. To facilitate the correct disposal of materials, the adoption of universally recognized recycling symbols has proven to be an effective strategy for organizing and guiding the collection and separation stages (Figure 3).

There are several recycling techniques, including mechanical, chemical, fluidization, and pyrolysis processes [17,19]. For plastic materials, the predominant methods are mechanical and chemical recycling, respectively classified as primary, secondary, and tertiary recycling [32]. Despite their advantages, these processes face environmental and economic limitations such as a high carbon footprint, elevated costs, and complexity in recovering polymers derived from material blends or contaminated polymers, as well as the incorporation of fibers and other additives that hinder efficient recycling [19,33].

Faced with these challenges, there is a growing movement toward sources of biobased plastics, with materials such as polylactic acid (PLA), polybutylene succinate (PBS), polyhydroxyalkanoates (PHA), and furano polyethylene (PEF), as well as others produced from starch, lignin, and agro-industrial residues. Despite advances, approximately 99% of plastics currently produced remain petroleum-derived polymers, which will continue to play an important role in various sectors for a long time [34].

In the packaging sector, glass stands out for being 100% recyclable and reusable without loss of quality, making it widely used due to its safety, chemical resistance, and ability to preserve the sensory attributes of foods and medicines. Glass recycling offers significant environmental benefits, such as reducing solid waste and conserving energy and raw materials. However, the high weight and logistical challenges related to transportation and delicate handling can hinder collection and full reuse of the material, limiting its expansion [19].

Paper and cardboard, increasingly popular as packaging materials due to their renewable and biodegradable nature, also stand out [35]. Recovered, laminated, or treated with resins, these materials improve their protective properties, although their susceptibility to humidity and water absorption still require improvements in manufacturing and treatment processes [19]. Recycling of paper and cardboard is a well-established practice that produces biomass useful for various applications. However, each recycling cycle reduces fiber length, which diminishes the strength of the final product and limits the number of reused cycles, typically six to seven times for high-quality paper. Therefore, there is a continuous need to improve destintaging and recycling processes to increase efficiency and enhance the sustainability of this cycle [35].

Multilayer packaging used in the food industry combines various materials, such as polymers, paper, aluminum, and organic or inorganic coatings. Despite their environmental advantages, especially when assessed through life cycle assessment (LCA), these solutions face a significant challenge: their high complexity makes recycling within the existing waste management infrastructure difficult [33,36]. Efficient separation of these multiple components remains limited, resulting in low reuse rates and contributing to waste that often ends up in landfills or is improperly discarded.

Metals such as aluminum, tinplate, and chromed sheets are widely used in food packaging due to their excellent barrier properties, strength, and versatility [19,37]. These materials can take various forms, ranging from rigid and semi-rigid packaging such as cans of different sizes and shapes, to bottles, tubes, caps, aluminum foils, or even in pulverized form, as in the metallization process of plastic packaging. This diversity enables a wide range of applications that meet the requirements for food preservation and transportation.

Metallic packaging offers advantages such as high resistance to light, gas, and humidity, as well as being highly recyclable and capable of being recycled indefinitely without loss of quality. Additionally, they allow easy molding into various shapes, support high temperatures for heating, feature a rigid structure that facilitates long-distance transportation, and enable unique decorating options, adding value to the final product. However, all these benefits come at a higher cost, both economically and environmentally. The production of metals, especially aluminum and steel, is associated with significant carbon dioxide emissions, contributing to global warming. Moreover, there are concerns regarding the leaching of toxic chemicals from metal containers and the depletion of non-renewable natural resources [37]. Therefore, although metallic packaging is highly efficient in many aspects, its use must be balanced with environmental and economic considerations, prompting the development of more sustainable alternatives and the improvement of recycling and manufacturing processes.

One of the most recent strategies for achieving sustainable actions was the creation of The European Packaging and Packaging Waste Regulation (PPWR), at the end of 2024. These rules, established by Regulation (EU) 2025/40, represent a significant update to the previous Directive 94/62/EC, aiming to promote a more effective and sustainable circular economy in the European Union.

This regulation introduces mandatory requirements for packaging design, aiming to ensure that all packaging is recyclable by 2030, based on Design for Recycling (DfR) criteria. Furthermore, it establishes progressive targets for recycled content in plastic packaging, requiring that at least 30% of the content of PET beverage bottles be recycled by 2030, increasing to 65% by 2040. The PPWR also provides for the implementation of reuse and refill systems, the ban on single-use plastic packaging from 2030, and the introduction of Extended Producer Responsibility (EPR) rates based on the recyclability performance of packaging. DfR assessments must consider parameters such as separability of components, sortability/recycling efficiency and yield, preservation of secondary-material functionality, and specific elements like additives, labels, sleeves, and closures so they do not hinder the recyclability of the main body [38].

Related to the requirements for substances in packaging is the presence of Substances of Concern (SoC) to be minimized, including considering adverse effects due to microplastics. Some metals and per- and polyfluoroalkyl substances (PFAS) used in food packaging must be under strict limits. For that, the entire chain involved must identify safer alternatives and validate their performance in real-life conditions, showing results with consistent documentation. On the other hand, for some specific items, like tea bags, fruit labels, and coffee pods, the rules require that they must be compostable. In this situation, the regulation mandates standardized consumer labeling to provide clear disposal guidance. In parallel, the stakeholders should explore technologies to facilitate automated sorting, such as AI-driven recognition systems [38,39].

These measures aim to reduce packaging waste, improve recycling quality, and increase the availability of recycled materials in the production chain. Technical documentation must demonstrate compliance, and all components must be compatible with established collection, sorting, and recycling processes.

PPWR is not limited to the European context, but tends to influence global practices, as the bloc is one of the largest consumer and export markets in the world. This requires multinational companies wishing to maintain access to the European market to adapt their products to design requirements for recycling, reuse, and packaging reduction. In this sense, the regulation will serve as a standardizer for global supply chains, encouraging other countries and regions to adopt similar legislation, both to harmonize trade and to advance more ambitious environmental goals. Thus, its implementation will accelerate the transition to circular economy models on a global scale [40].

## 3. Sustainable Innovations for Food Packaging

Food products have a limited shelf life, which can be related to specification characteristics, properties, and storage conditions of the products. Environmental factors, such as light exposure, oxygen levels, temperature, and moisture, can accelerate food decomposition, thereby reducing the product’s shelf life [41,42]. However, selecting appropriate packaging materials can enhance the shelf-life characteristics of these products [43].

**Figure 4:**
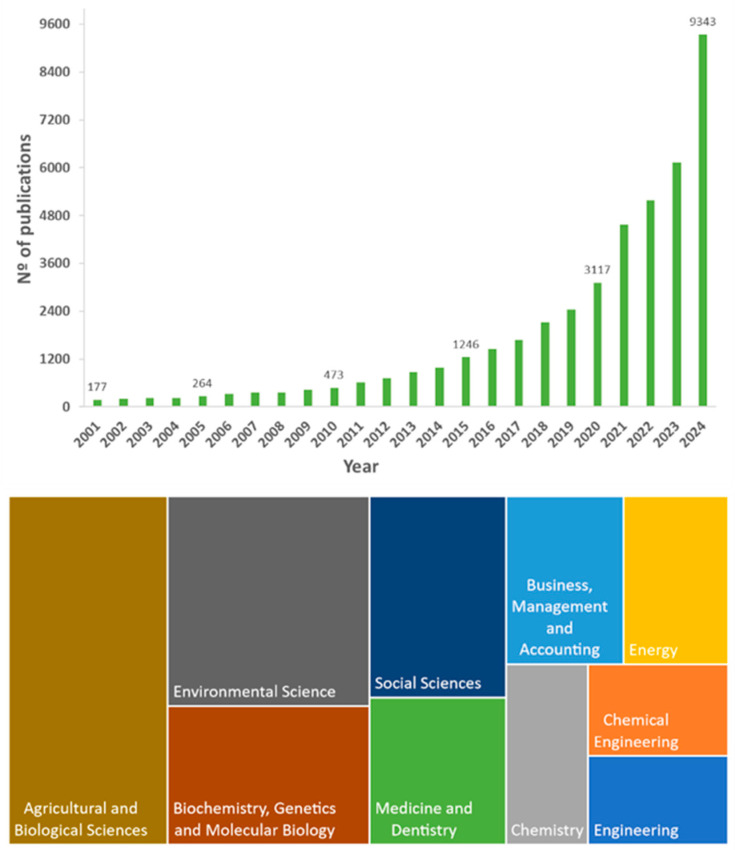
Number of review and research articles published, plotted against year of publication and application areas of food packaging innovation over 2001–2024. (Source: www.sciencedirect.com (accessed on 15 June 2025); keywords: food packaging innovation).

Recent scientific publication trends indicate increased interest in food packaging innovation (Figure 4). In addition to scientific studies in the area, analyzing and understanding alternative biobased material sources is a critical tool for directing efforts toward the development of innovative technologies. Currently, this knowledge is crucial for enhancing anticipatory capacity and stimulating the organization of innovation systems within the academic environment [23,24,44].

Petroleum-based materials fulfill several essential requirements for food packaging quality; they are inexpensive, transparent, durable, flexible, easily processable, and offer higher mechanical and barrier properties against moisture, oxygen, carbon dioxide, and contaminants [3,45]. All of these characteristics suggest that petroleum-based packaging is highly effective in preserving fresh food, increasing shelf life, and ensuring product and consumer safety [46,47].

**Figure 5:**
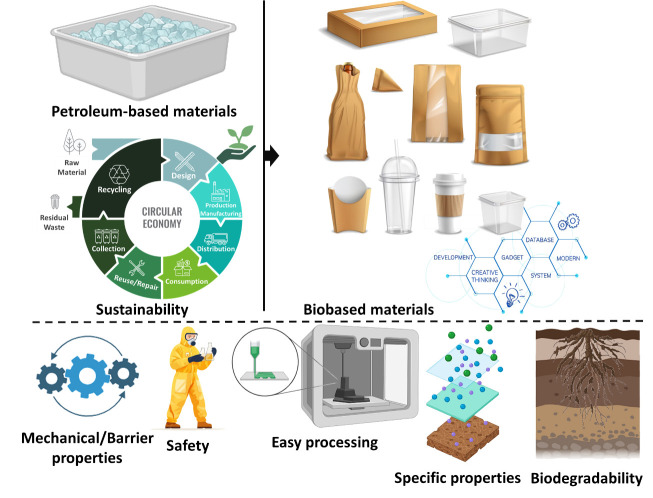
Important factors in choosing biomaterials for food packaging.

However, the overreliance on petroleum-based materials, combined with food demand, population growth, changes in consumption and disposal patterns, inefficient waste management practices, and other complex factors, has resulted in substantial environmental issues, causing severe global environmental and climate changes, resource depletion, and greenhouse gas emissions. Thus, to mitigate environmental challenges, biodegradable materials sourced from renewable resources, abundant in nature, cost-effective, non-toxic, biodegradable, and bearing the potential to incorporate novel functionalities have emerged as a sustainable and eco-friendly alternative (Figure 5) [24,48,49,50].

Bioplastics are a potential innovation material promoting sustainable development in several food packaging areas. Bioplastics can be defined as plastics that are either biobased or biodegradable, with properties similar to those of petroleum-based conventional plastics while offering additional advantages, such as a reduced carbon footprint, enhanced functionalities, and alternative waste management options, including organic recycling [51,52,53]. These sustainable advancements are responsible for the development of new or enhanced processes, products, services, organizational methods, and marketing strategies that significantly mitigate negative impacts or enhance positive environmental, social, and/or economic outcomes.

**Figure 6:**
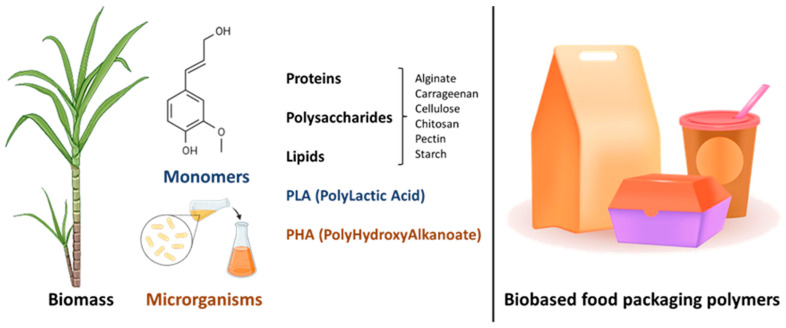
Most common biobased polymers available on the market.

Biobased food packaging materials are essential to mitigate the impacts of packaging and food waste and the environmental, social, and economic impacts. Thus, the development of biopolymers and edible and biodegradable films is an important effort [54,55]. Based on their raw material sources, these biopolymers can be classified as natural, synthetic, or microbial biopolymers (Figure 6) [50].

Natural biopolymers are extracted from polysaccharides, lipids, and proteins, including cellulose, alginate, starch, chitosan, pectin, gum, carrageenan, seaweed, gluten, and derivatives [56,57,58,59,60,61,62]. Synthetic biopolymers are usually made through chemical processes from biological monomers and consist of aliphatic-aromatic copolymers, aliphatic polyesters, polylactides, and aliphatic copolymers, using renewable biobased monomers like poly (lactic acid) (PLA) and oil-based monomers like polycaprolactone. Microbial biopolymers are produced by microorganisms or genetically modified bacteria, i.e., polyhydroxyalkanoates (PHA), a polyester synthesized through bacterial fermentation of sugars and lipids [50,63,64,65].

Nowadays, several biobased polymers are available on the market, including PLA, PHA, Poly (butylene succinate) (PBS), thermoplastic starch (TPS), partially biobased poly (ethylene terephthalate) (bioPET), bio-polyethylene (bioPE), soybean-based, cellulose-based, and others [22,66,67]. The poor mechanical, barrier, and processability behaviors, hydrophilic characteristics, brittleness, and limited long-term stability properties are the most important disadvantages of these biopolymers, limiting their use in food packaging despite their biocompatibility, degradability, availability, and thermoplastic characteristics [55,66,68].

Bioplastics derived from biobased materials can present enhanced life cycle performance and improved biopolymer properties through physical blending with organic acids, plant waste extracts, essential oils, synthetic carbonyl, natural fibers, and other composites. Also, food waste, such as potatoes, fish, poultry, fruit, and vegetables from processing, has been extensively considered to enhance the properties of these biopolymers [50,67,69].

Thus, sustainable food packaging is undergoing a transformative evolution. Moreover, two other important innovations can be considered, such as the development of advanced biopolymer nanocomposites and the integration of artificial intelligence (AI) [70,71,72]. These advancements are propelling a shift towards smart packaging systems and data-driven material optimization, ultimately aiming to improve functionality, ensure food safety, and achieve greater environmental sustainability [73,74,75].

Advanced biopolymer-based nanocomposites significantly enhance packaging performance by improving barrier properties, mechanical strength, and antimicrobial activity while retaining biodegradability. Multifunctional films derived from agricultural waste support circular economy models through improved food preservation. Additionally, nanotechnology enables the development of edible and active packaging with embedded bioactive compounds that extend shelf life and reduce food waste [70,74,76,77]

While the development of advanced biopolymer-based nanocomposites addresses the critical need for materials with enhanced barrier and functional properties, their full potential can be highlighted through AI-driven informatics for material selection and design optimization [78,79]. These computational tools are indispensable for navigating the complex multi-objective trade-offs, enabling the creation of tailored packaging solutions that optimally balance sustainability, cost, and functionality [80,81]. This synergy also extends into operational intelligence, enhancing full-chain traceability and allowing for the accurate prediction of shelf life. Thus, these capabilities foster a truly responsive and sustainable packaging ecosystem [80,82,83].

This is one of the most important strategies of circular economy practices, reducing the consumption of virgin raw materials and minimizing waste generation, converting these waste materials generated under the linear economy model into valuable resources through processes such as regeneration, restoration, and renovation, promoting a sustainable and resource-efficient system [49,84,85].

Thus, the transition to a circular economy can improve the life cycle of products and processes within the food packaging sector, driving sustainability and efficiency [85,86,87]. During this transition, to achieve sustainable progress and alignment with a circular economy, the key sectors of the production and consumption chain should be committed to developing innovative packaging solutions [48,84,88,89].

## 4. Applications of Carbon Compounds in the Development of Functional Packaging

Nanotechnology in food packaging has enabled significant advancements by incorporating active and intelligent functionalities that enhance microbiological safety and extend product shelf life [90] In this context, recent studies have shown that incorporating carbon nanomaterials into polymer matrices, whether synthetic or biological, is a promising strategy for developing food packaging [91,92,93,94]. This combination allows for high functional performance while reducing environmental impact, driving the development of active and intelligent systems capable of providing antioxidant stability, antimicrobial activity, and quality monitoring during storage [91,95]. Furthermore, these nanomaterials exhibit good compatibility with various polymers, significantly enhancing mechanical and barrier properties, especially protection against UV radiation [96]. Among the most studied for application in food packaging are carbon nanotubes (CNTs), carbon quantum dots (CQDs), and carbon nanofibers (CNFs), which are being explored in different polymer matrices.

The incorporation of CNTs into active films developed from polylactic acid (PLA), poly (ε-caprolactone) (PCL), and cinnamaldehyde (CIN) resulted in a 25.5% increase in tensile strength and a 28.4% rise in the elastic modulus of the films. These improvements were attributed to the surface area of the CNTs, their nanometric dispersion within the polymer matrix, and the good compatibility between the CNTs and PLA. In addition to enhancing mechanical properties, the presence of CNTs contributed to increased resistance to ultraviolet radiation and higher absorbance values in the visible region. Another relevant aspect was that the CNTs/PLA system acted as a controlled release system for CIN, reducing evaporation and extending its antimicrobial activity. As a result, the films demonstrated effective activity against *Staphylococcus aureus* and *Escherichia coli*, confirming their potential for use in active food packaging applications [97].

Another study explored the use of carbon quantum dots obtained from dried lemon peel (LCQDs) incorporated into polyvinyl alcohol (PVA) films, where it was observed that the high compatibility between LCQDs and the polymer matrix contributed to the improvement of mechanical and barrier properties, especially when added at higher concentrations (3%). These films showed complete UV radiation blocking (100%), a high antioxidant capacity, and significant antimicrobial activity against *S. aureus*, achieving the largest inhibition zone (27 mm), as well as effectiveness against *Salmonella enterica*, *E. coli*, *Bacillus cereus*, and *Listeria monocytogenes* [98]. The results underscore the potential of agro-industrial residues as renewable and sustainable sources of carbon nanomaterials for application in active food packaging.

Similarly, carrageenan-based films containing zinc-doped carbon quantum dots (Zn-CDs) and anthocyanins from kohlrabi peel also exhibited multiple functions, including significant UV blocking (85.2% for UV-A and 99.4% for UV-B), high antioxidant activity (99% in the ABTS assay and 58.6% in DPPH), and strong antimicrobial action against the growth of Gram-positive and Gram-negative bacteria. Furthermore, when applied to shrimp, these films were capable of visually monitoring freshness, delaying deterioration, and extending the product’s shelf life, combining preservation functionalities with intelligent packaging features [99]. In meat packaging, composite films made of chitosan and starch incorporated with nitrogen- and phosphorus-doped carbon quantum dots (NP-CDs) showed significant performance when applied directly to fresh meat, as the films effectively reduced bacterial growth to less than 2.5 log CFU/g after 48 h of storage at 20 °C, without altering the appearance of the product, demonstrating their potential as a safe and efficient preservation technology [100].

CNFs have also been receiving increasing attention as a functional material in food packaging. When incorporated into calcium-crosslinked sodium alginate films, even at low concentrations (0.1% *w*/*w*), CNFs promoted a significant enhancement of antimicrobial and antiviral activity, inhibiting *S. aureus* and showing action against non-enveloped viruses. These effects were achieved without compromising biocompatibility, as confirmed by the absence of cytotoxicity in human HaCaT cells [101].

**Figure 7:**
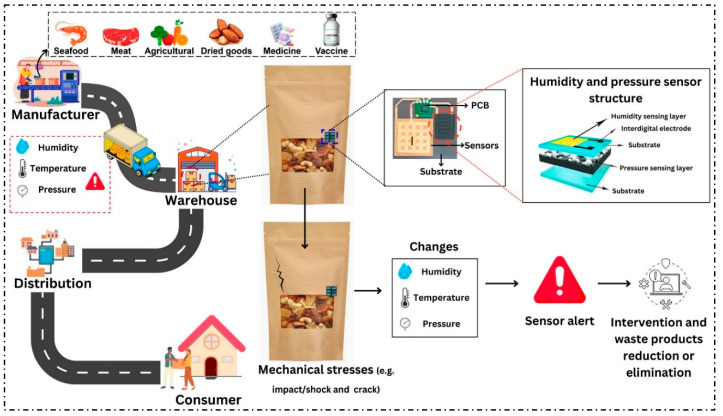
Schematic diagram of the use of carbon sensors in smart packaging for environmental variable monitoring and loss reduction. Adapted with permission from Guo at al. (2025) [102]. Copyright 2025 Creative Commons CC BY 4.0 license.

In addition to their use in nanocarbon-based composites aimed at enhancing performance and functional properties, their application in the development of sensors for smart packaging is also noteworthy [102]. As illustrated in Figure 7, these devices can be integrated into packaging to continuously monitor environmental variables such as humidity, temperature, and pressure throughout the supply chain, including production, storage, and distribution. This enables the detection of mechanical failures (such as impacts and cracks), real-time alert generation, and the facilitation of rapid interventions to minimize losses and reduce food waste.

Overall, recent advances confirm that carbon-based materials have great potential to revolutionize the food packaging sector, from structural reinforcement and the addition of active functionalities in polymer films to the development of fully carbon-based smart sensors. These results demonstrate that carbon-derived nanomaterials not only extend shelf life and increase microbiological safety of perishable foods but also contribute to the consolidation of active and intelligent packaging solutions aligned with the demands for sustainability and technological innovation in the food industry.

## 5. Key Challenges in Sustainable Innovations for Food Packaging

Currently, climate change and sustainable development represent the biggest and most critical global issues demanding increased attention and several efforts. Recognizing these emerging topics and areas of development, as well as tracking key research trends, are the initial steps toward creating innovative research materials [51,52,103].

Recently, the development of new, advanced, and sustainable materials has been one of the most important interests of science and technology, along with consumer demand and the mitigation of climate change [24,52,103,104]. Therefore, effective and sustainable packaging design extends beyond its primary protective function. It constitutes a complex, multi-disciplinary process which demands collaboration among all sectors of the supply chain, with specific alignment to the intended market’s infrastructure and consumer behavior [4,105,106].

Thus, the environmental impact of packaging is related to the properties of the food products, including their perishability, cost, emissions, and resource depletion during the production processes [4,107]. The enhancement of food packaging design is crucial to improve the overall sustainability profile by reducing the environmental impact of both the packaging material and the food product through minimized end-of-life waste [3,4].

Furthermore, global food waste can be related to postharvest activities, processing, transportation, storage, and inadequate packaging. Innovations throughout the entire supply chain are essential, ranging from raw material sourcing to food processing, quality assurance, and packaging [24,104,108].

Plant-based bioplastics, including both biodegradable (e.g., PLA, PHB) and non-biodegradable (e.g., bio-PE, bio-PP), are increasingly commercialized. Innumerable studies are also utilizing agri-food waste as feedstock to reduce agricultural impacts [109,110]. For non-biodegradable materials, effective recycling within existing infrastructure depends on enhanced recyclability, high retrieval rates, and efficient separation within waste streams [4,105].

Global bioplastics production will increase from around 2.2 million tonnes in 2022 to 6.3 million tonnes by 2027 [111]. Around 48% (1.07 million tonnes) and 43% (934,000 tonnes) of bioplastic production was for the packaging sector in 2022 and 2023, respectively [46,112]. The growing commercial acceptance of bioplastics reflects an increasing commitment to environmentally sustainable practices among both consumers and producers. However, the reduced bioplastics on the market, around 0.5% of global production, further underscores the existing challenges and emphasizes the necessity for greater adoption, investment, and scalability to achieve meaningful environmental impact [113,114].

The most significant challenges to sustainable innovations in food packaging can include high costs, significant scalability challenges, technical limitations, regulatory barriers, and fragmented stakeholder collaboration, decreasing competitiveness [115,116,117]. Thus, sustainable materials often exhibit inferior performance in terms of barrier properties and durability, which limits their effectiveness [116,118,119]. Furthermore, the biodegradable and compostable packaging end-of-life often requires specific waste management infrastructure, which is lacking in many countries. The absence of appropriate waste management systems generates consumer uncertainty, leading to the incorrect disposal of these materials. Consequently, the lifecycle environmental benefits are critically compromised, creating a formidable impediment to the widespread adoption of these packaging solutions [54,120,121].

Furthermore, a critical gap remains in the development of interdisciplinary frameworks that identify the essential economic, social, and environmental systems influencing bio-based transformation processes and their outcomes [122,123]. Advancing the bioeconomy requires a deeper understanding of the factors that drive the emergence of key enabling technologies, as well as the development of tailored policy incentive schemes that can effectively promote sustainable innovation under specific contextual conditions [124,125]. This approach provides a forward-looking strategy that aligns economic development with sustainable resource management by fundamentally transforming the valuation and application of bio-based renewable resources [122,123,124,125].

Another significant challenge to biobased transition is the management of natural resources such as water and biomass production, requiring an increase of around 54% of the current corn production and 60% of annual freshwater withdrawal [122,126,127,128].

Biopolymer-based materials present limitations such as high production costs, poor mechanical properties, inconsistent biodegradation rates, competition with food resources, and difficulties in recycling processes [112].

In the food packaging sector, selecting materials that ensure safety, integrity, and prolonged shelf life is crucial. Key factors to consider include chemical-physical, thermal, mechanical, and barrier properties, as well as optical characteristics [11,129]. This transition requires expensive raw materials, implementation of downstream recovery and recycling processes, chemical compatibility, and end-of-life studies [67,114,130]. However, food waste presents higher potential as a carbon feedstock for biobased materials and cost-effectiveness, reducing bioplastic production costs [130,131,132].

## 6. Consumer Acceptance

The current landscape is characterized by a duality regarding the use of packaging and sustainability. While accelerated consumption, mainly driven by the expansion of digital commerce, contributes to a significant increase in waste generation, society is increasingly aware of environmental impacts [133]. This contrast highlights the challenge of balancing convenience and practicality with the urgent need to rethink the use of natural resources and adopt more responsible practices throughout the consumption chain [134].

Acceptance of sustainable food packaging is strongly influenced by personal attitudes and individual values related to environmental responsibility [135]. Emotional and ethical elements play a central role in how consumers perceive this type of packaging, demonstrating that engagement goes beyond rational factors [136]. In this regard, marketing and communication strategies that emphasize environmental benefits and companies’ commitment to sustainable practices can be decisive in increasing acceptance and adoption of these solutions in the market [137].

A study conducted with consumers in Vietnam identified three core dimensions shaping perceptions of eco-friendly packaging: materials used, manufacturing technology, and market appeal. Consumers associate sustainable packaging with biodegradable or recyclable materials, assigning lower environmental value to plastics, despite recognizing their superior performance in food protection. Limited understanding of the technological processes involved in production was also observed, reinforcing the need for greater transparency and education about the packaging lifecycle. Additionally, attributes such as attractive visual design, functionality, and competitive price were considered key factors for acceptance, with cost being a significant barrier [138]. The study suggests that manufacturers and public policies consider these perceptions to expand the adoption of environmentally responsible packaging solutions in the packaged food market.

Another research study conducted in the United Kingdom indicated that most participants were willing to recycle and showed good adherence to separating plastic trays. The research also highlighted knowledge gaps regarding the recyclability of certain materials, such as black trays and compostable packaging. Confusion about proper disposal procedures and label reading indicates the need for clearer guidelines and educational campaigns. Despite these limitations, the study identified an engaged and environmentally conscious consumer profile, mainly composed of highly educated women with employment or student ties, which may have positively influenced the results [139]. Still, the data underscore the potential for expanding the use of sustainable packaging, provided that accessible and clear information is available to consumers.

Additionally, recent studies have focused on how consumers perceive and accept sustainable innovations in packaging, including edible coatings and nanoparticle-based technologies [140]. These studies indicated that acceptance varies depending on the generation, profession, and geographic location of respondents. Younger individuals, especially from Generation Z, and those involved in the food sector showed greater willingness to purchase products with sustainable packaging. Regional differences were also observed, with Mexican consumers demonstrating higher acceptance. Another study explored factors influencing the acceptance of edible packaging, revealing that consumer innovativeness positively impacts perceptions of this type of packaging, increasing attitudes, subjective norms, and perceived control over purchasing behavior. However, attitudinal ambivalence was identified as a moderator, dampening or amplifying these relationships, serving as an indicator of the psychological mechanisms behind the adoption of sustainable packaging [141].

Growing awareness and consumer demands directly influence companies’ decisions, steering the packaging industry toward more sustainable solutions and promoting significant advances in material selection, responsible design development, and environmentally conscious practices [142,143]. Interviews with professionals in the food packaging industry in countries such as Germany, Austria, Spain, and Portugal revealed differing perceptions regarding the importance of sustainability for consumers. Half of the interviewees believed consumers do not value sustainability criteria in packaging, focusing solely on sourcing materials, such as a preference for paper over plastic. Additionally, these professionals showed a lack of awareness regarding evidence of consumer interest in attributes like biodegradability, recycled content, and bio-based materials [144].

Given the different perspectives surrounding packaging sustainability—from consumer perceptions to industry viewpoints—it is clear that the transition to environmentally responsible solutions requires a joint effort. While consumers demonstrate increasing interest in sustainable attributes such as biodegradability, recyclability, and innovation, they still face barriers related to information, ambivalence, and cost. Conversely, the industry does not always recognize this engagement, which can limit the adoption of more conscious practices.

## 7. Conclusions

The literature review revealed that the development of sustainable packaging represents a fundamental commitment to reducing environmental impact and advancing the circular economy. The adoption of sustainable practices still faces challenges, primarily due to some industries’ resistance to investing in solutions that go beyond marketing. Many companies continue to see sustainability as a competitive advantage rather than a necessity, which limits the long-term positive impact of their actions.

Recyclable, reusable, biodegradable, and compostable packaging options offer alternatives with the potential to reduce waste and promote sustainability within the packaging sector. However, the implementation of these solutions still encounters significant obstacles, such as consumer awareness, supply chain adjustments, and the development of infrastructure for reverse logistics and composting. Additionally, the high production costs of these alternative materials can hinder widespread adoption, especially for small and medium-sized enterprises. For a successful transition to these packaging solutions, concerted efforts among industry, governments, and society are essential to maximize economic, social, and environmental benefits.

Another crucial factor for the evolution of sustainable packaging is the promotion of research and development of new materials and processes. The use of bioplastics, nanomaterials, and other technological innovations has demonstrated great potential to reduce the ecological footprint of packaging while ensuring protection and safety for the packaged products. Equally important is the active participation of consumers in this change.

Despite progress made, significant challenges remain, such as regulatory complexity, economic viability of new technologies, and the need for consumer education to foster acceptance of innovative solutions. These challenges highlight that the journey toward a sustainable packaging sector is a collective and ongoing effort. Therefore, it is essential for companies, governments, and consumers to work together to overcome these barriers and promote solutions that balance convenience with sustainability.

## Data Availability

No new data were created or analyzed in this study. Data sharing does not apply to this article.
